# Transient Network at Large Deformations: Elastic–Plastic Transition and Necking Instability

**DOI:** 10.3390/polym8040108

**Published:** 2016-03-24

**Authors:** Fanlong Meng, Eugene M. Terentjev

**Affiliations:** Cavendish Laboratory, University of Cambridge, Cambridge CB3 0HE, UK; fm437@cam.ac.uk

**Keywords:** transient network, large deformation, elastic–plastic transition, necking instability

## Abstract

We theoretically investigate the mechanical response of a transient network, which is characterised by dynamically breaking and re-forming crosslinks, and accounts for the finite chain extensibility (thus permitting the large deformations to be described). We build the general theory that incorporates the widely accepted empirical model of hyper-elasticity at large deformations (the Gent model) and naturally includes the microscopic behavior of transient crosslinks under the local tension applied to them. The full analytical expression for the elastic energy, or equivalently, the constitutive relation for arbitrary deformation is derived, and then the example of uniaxial tensile strain is focused on. In this case, we show that the mechanical response depends on the ratio of the imposed strain rate and the breakage rate of the crosslink: the system flows plastically (over a yield point) when the strain rate is much smaller than the breakage rate, while it remains elastic when the strain rate is much larger than the breakage rate. There is a broad range of this transition when the elastic and plastic regions of the sample coexist, and a resulting necking instability occurs. As a generalisation, we also consider a dual transient network, with two components penetrating each other, each having its own microscopic crosslink dynamics. The two networks add their local forces and share the deformation; we find that the network with a lower breakage rate determines the global deformation of the system.

## 1. Introduction

**Figure 1:**
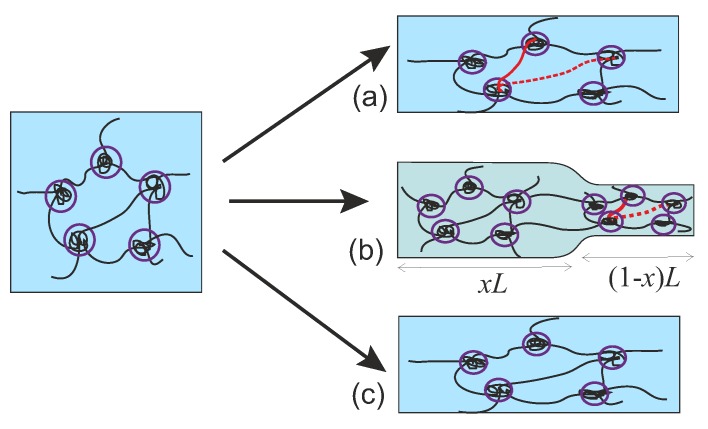
A transient network: (**a**) responds with a plastic flow under slow deformation; (**b**) forms a characteristic neck connecting the elastic (x) and plastic (1-x) regions under a deformation with a moderate rate; and (**c**) behaves like an elastic rubber under a high rate of deformation. In (**a**,**b**), the red dashed line represents a chain broken from a crosslink, and the solid red line represents the chain re-crosslinked at a new site.

When a transient or dynamically crosslinked gel is uniaxially stretched as schematically shown in Figure 1, it can behave as a plastically flowing melt upon a slow rate of deformation, as an elastic rubber under a fast rate of deformation—or it may enter an intermediate regime of coexistence when a neck is formed, connecting a highly and a weakly deformed region in the sample. Moreover, due to the ability of transient crosslinks to break and re-connect in the network, the above processes can be repeated for many cycles without inducing any permanent damage to the material. Materials with such self-healing properties have become attractive for many applications in biological substitutes, bio-compatible sensors, drug delivery, and various industrial settings, as reviewed in [1,2,3,4].

All of these self-healing materials have a common feature: crosslinks in the network can be dynamically broken and reformed. The dynamic crosslinks, mainly categorized into physical ones and chemical ones, can be obtained in various ways. Physically, the chains can be crosslinked via hydrophobicity [5,6,7], hydrogen bonding [8,9], ionic interaction [9], crystallization [10,11,12], *etc.* This class of materials is quite commonly used in what is called “shape memory” plastics, where a given shape of sample may be (re)recorded by plastic deformation at high temperature, and fixed by the physical (re)crosslinking [12,13,14]. Chemically, crosslinking of polymer chains can be achieved in forms of disulfide bonding [7], imine bonding [15], reversible radical reaction [16], *etc*. Recently, there was a new type of transient network reported, named “vitrimer” [17,18,19], in which chemical groups of polymer chains exchange via transesterification reaction or transamination of vinylogous urethanes.

A transient network under small deformations is well studied theoretically, from either a microscopic [20,21,22,23,24] or a macroscopic viewpoint [25,26], where the crosslinks are dynamically broken and reformed all the time. Such theories are valid when the material undergoes a sufficiently small deformation, so the constituting chains remain Gaussian, but cannot be accurate for large deformations when the network chains may be stretched significantly past the Gaussian limit (exploring their finite extensibility). Normally, in models of rubber elasticity, the finite extensibility regime is not very important because most of the relevant physical effects occur (and are well-described) within the limit of Gaussian network chains. However, due to a possibility of plastic flow in the transient network, the regime of large chain extension can be reached rapidly, and so the theory needs to incorporate the finite-extensibility limit. Until now, only a few theoretical studies have looked at a transient network under a large deformation. Wang and Hong [27] proposed a macroscopic theory for a dual networks where one transient network (with no ability to re-connect broken crosslinks) and one permanent network penetrate each other under a finite deformation, using the Gent model [28] as the continuum elasticity model for finite deformations. This work was later developed by Long and Hui [29,30] by adding the ability of crosslinks to re-connect. However, neither work allowed for the plastic flow, since the second interpenetrating network remains permanent and elastic all the time. Moreover, in these studies, the dynamics of transient crosslinks is treated empirically by assuming an expression for the evolution of the crosslinks in the network, with several phenomenological parameters to be fitted to experiments. As a result, necking instability is successfully predicted by them. However, compared with the much detail and success in explaining the response of the transient network under a small deformation, the understanding on how a transient network responses to a “finite deformation" is still relatively poor.

In this work, we will bridge the macroscopic deformation of the whole network and the microscopic dynamics of the crosslinks in a unified framework by assuming affine deformation of the network. Macroscopically, we will use the Gent model for finite rubber elasticity to describe the deformed energy of the transient network when its reference state is dynamically changing. Microscopically, the breakage and the reforming of the crosslinks depend on the local forces exerted on the polymer chains, which can be obtained from the continuum energy density. By applying this general model, we will discuss how a transient network deforms when undergoing a uniaxial extension, which is a traditional testing geometry in rheology. As an extension, a dual transient network will also be studied at the end of this paper.

## 2. Formalism for a Single Transient Network

### 2.1. Rates of Reactions

We will briefly describe how the crosslinks behave in a deformed polymer network, mainly following the ideas developed by Tanaka and Edwards [31]. In order to be thermally broken from a crosslinking bond (junction), the chain must overcome an energy barrier, $W_{b}$, which can originate from the physical interactions or the chemical bonding with another chain at the same site. If a polymer chain is in its force-free state, then the breakage rate of a crosslink can be defined by a thermal activation law:

(1) $$\begin{array}{ccc} \beta_{0} & = & \omega_{0}e^{-W_{b}/k_{B}T}, \end{array}$$

where $\omega_{0}$ is the frequency of thermal vibrations of the reactive group in an isolated state. If there is a force, f, exerted on the chain at time *t*, which was crosslinked at time $t_{0}$, then the breakage rate at time *t* will become:

(2) $$\begin{array}{ccc} \beta (t;t_{0}) & = & \beta_{0}e^{fb/k_{B}T}, \end{array}$$

where *b* is the size of an individual chain segment, which is considered as the distance for a chain to be broken from a crosslinking bond. The force in Equation (2) depends on both the current time *t* and the time $t_{0}$ when it was crosslinked, and the explicit expression of it will be provided later.

If the diffusion time for a dangling chain to find its next crosslinking site is very short, then the reaction rate for crosslinking can be expressed by

(3) $$\begin{array}{ccc} \;\rho_{0} & = & \omega_{0}e^{-W_{c}/k_{B}T}, \end{array}$$

where $W_{c}$ is the energy barrier for a dangling chain to be bonded with a new crosslinking site. Usually, the re-crosslinking barrier is much smaller than the breakage one at ambient temperatures, $W_{c}\ll W_{b}$, resulting in a much larger rate for re-crosslinking, $\rho_{0}\gg \beta_{0}$, and a stable fully crosslinked network. At very high temperatures, the re-crosslinking and the breakage rate can become comparable with each other, when $\rho_{0}\approx \beta_{0}\approx \omega_{0}$. In practice, the diffusion time $t_{d}$ for a dangling chain to become relaxed and find its re-crosslinking site can be significant, especially a reptation process and tube-constraint release are required, so the actual re-crosslinking rate would become smaller correspondingly. As in other combined reaction-diffusion systems, the effective re-crosslinking rate can be expressed by a combination of two limits:

(4) $$\begin{array}{ccc} \;\rho & = & \frac{\rho_{0}}{1+\rho_{0}t_{d}}. \end{array}$$

### 2.2. Evolution of Crosslinked Chain Populations

Suppose $N_{c}\left(t\right)$ and $N_{b}\left(t\right)$ are the number of the crosslinked and the dangling chains at time *t*, respectively, with the total number of chains a constant: $N_{\mathrm{tot}}=N_{c}\left(t\right)+N_{b}\left(t\right)$. The network is assumed to be at its force-free state at time t=0, and deformed at time t>0 with the deformation tensor E(t;0) that may change with time. After an infinitesimal time step Δt, the total number of the crosslinked chains will become $N_{c}\left(0\right)\left[1-\beta \left(\Delta t;0\right)\Delta t\right]+N_{b}\left(0\right)\rho \Delta t$, where the first term represents the number of crosslinked chains surviving from t=0, and the second term represents the number of chains newly crosslinked during the time interval from t=0 to t=Δt. Since the time step is negligibly small, (1-β(Δt;0)Δt) can be replaced by exp[-β(Δt;0)Δt]. Then, after another time interval Δt, the number of crosslinked chains will become $N_{c}\left(0\right)e^{-\beta (\Delta t;0)\Delta t-\beta (2\Delta t;0)\Delta t}+N_{b}\left(0\right)\rho \Delta te^{-\beta (2\Delta t;\Delta t)\Delta t}+N_{b}\left(\Delta t\right)\rho \Delta t$, where the first term represents the number of the crosslinked chains surviving from t=0 to t=2Δt, the second term represents the number of surviving crosslinked chains which were crosslinked at time Δt, and the third term represents the number of chains newly crosslinked during the time interval from t=Δt to t=2Δt. Repeating the above steps, the total number of crosslinked chains after a time NΔt can be written as $N_{c}\left(0\right)e^{-\sum_{i=1}^{N}\beta \left(i\Delta t;0\right)\Delta t}+\sum_{j=0}^{N-1}N_{b}\left(j\Delta t\right)\rho \Delta te^{-\sum_{k=j+2}^{N}\beta \left(k\Delta t;\left[j+1\right]\Delta t\right)\Delta t}$. If we convert this into the continuous form, the total number of the crosslinked chains at time *t* can be written as:

(5) $$\begin{array}{c} N_{c}\left(t\right)=N_{c}\left(0\right)e^{-\int_{0}^{t}\beta \left(t^{\prime };0\right)dt^{\prime }}+\int_{0}^{t}\rho N_{b}\left(t^{\prime }\right)e^{-\int_{t^{\prime }}^{t}\beta \left(t^{\prime \prime };t^{\prime }\right)dt^{\prime \prime }}dt^{\prime }, \end{array}$$

where the first term on the right hand side represents the number of the crosslinked chains surviving from time t=0 to the current time *t*, and the second term represents the number of the crosslinked chains surviving from their new crosslinking time $t^{\prime }$ to the current time *t*.

### 2.3. Hyper-Elastic Energy at Large Deformations

Due to the dynamical nature of crosslinks, the reference (or the force-free) state, of the network can only be defined dynamically and locally, depending on when the crosslinks are broken and reformed. In this sense, a transient network can be regarded as a permanent network, but with a dynamically changing reference state. Thus, here we first introduce the free energy form of a permanent network under a finite deformation, before discussing how it changes in the transient case.

There are many models for describing the elasticity of a rubbery network, and the free energy density of the material $F_{\mathrm{pe}.n.}$ can be very different [32]. For a permanently crosslinked network undergoing a finite deformation, the Gent hyperelastic model is widely regarded as most successful in matching the experiment [28,33]. It also has the advantage of being extremely analytically simple. If a reference state is defined at time t=0 and the material is deformed with a tensor E(t;0) at time *t*, then the free energy density of the network from the Gent model can be written as:

(6) $$\begin{array}{ccc} F_{\mathrm{Gent}}\left(t;0\right) & = & -\frac{1}{2}GJ_{m}\ln\left(1-\frac{J(t;0)}{J_{m}}\right), \end{array}$$

where *G* denotes the linear rubber shear modulus, the scalar parameter $J_{m}$ defines for the finite stretchability limit of the network, and J(t;0) is the first strain invariant defined as

(7) $$\begin{array}{ccc} J(t;0) & = & \mathrm{tr}\left[E\left(t;0\right)E^{T}\left(t;0\right)\right]-3=\sum_{i}\lambda_{i}^{2}-3, \end{array}$$

where $\lambda_{i}^{2}$ are the eigenvalues of the Cauchy deformation tensor, $E\left(t;0\right)E^{T}\left(t;0\right)$, and $\lambda_{i}$ themselves are the stretching ratios along three principle directions. When $J_{m}$ approaches infinity (*i.e.*, the limit of chain extensibility is not explored), the Gent model will reduce to the classical rubber elasticity (Gaussian or neo-Hookean model) $F_{\mathrm{nH}}\left(t;0\right)=\frac{1}{2}GJ\left(t;0\right)$. Alternatively, the neo-Hookean model is useful for handling the network at small relative deformation at $J\ll J_{m}$.

As mentioned before, the rate for a chain to break from a crosslink is a function of the local force exerted on it. In a network with the crosslink density *n* and the average mesh size $r_{i0}$, the local force acting on a chain at time *t* , which was crosslinked at time $t_{0}$, can be obtained from the engineering tensile stress $\partial F/\partial \lambda_{i}$ and the average cross-section area of the “mesh cell”:

(8) $$\begin{array}{c} f_{i}\left(t;t_{0}\right)=\frac{1}{nr_{i0}}\frac{\partial F(t;t_{0})}{\partial \lambda_{i}}\;=\frac{1}{nr_{i0}}\frac{GJ_{m}}{J_{m}-J\left(t;t_{0}\right)}\lambda_{i}\left(t;t_{0}\right), \end{array}$$

where the second expression is obtained by applying the Gent model as the free energy density. When the stretchability $J_{m}$ approaches infinity, *i.e.*, in the neo-Hookean model limit, the force in Equation (8) reduces to $f_{i}\left(t;t_{0}\right)=3k_{B}{Tr}_{i}\left(t;t_{0}\right)/Nb^{2}$, where *N* is the number of monomers connecting two crosslinks, which is exactly the expression for a Gaussian chain. In a transient network, the reference state can only be defined locally and dynamically, as its structure changes with time. Consider the geometry of uniaxial stretching as an example. If a chain is crosslinked at time *τ*, when the whole system is stretched with a stretching ratio λ(τ) (making this the reference state for this chain), then the corresponding force exerted on the chain at a subsequent time *t*, when the whole system is stretched with a stretching ratio λ(t), can be obtained by the orientational averaging of the force on a crosslinked strand, assumed isotropically distributed in 3D. The integral over angles is calculated analytically and, using Equation (8), we obtain:

(9) $$\begin{array}{ccc} \langle f(t;\tau )\rangle & = & \frac{1}{nr_{i0}}\frac{GJ_{m}}{J_{m}-J\left(t;\tau \right)}\int_{0}^{\pi /2}d\theta \sin\theta \sqrt{{\left(\frac{\lambda (t)}{\lambda (\tau )}\right)}^{2}\cos^{2}\theta +\frac{\lambda (\tau )}{\lambda (t)}\sin^{2}\theta }\\ & = & \frac{1}{2nr_{i0}}\frac{GJ_{m}}{J_{m}-J\left(t;\tau \right)}\sqrt{\frac{\lambda (\tau )}{\lambda (t)}}\left(\sqrt{1+a}+\frac{\text{ArcSinh}\left[\sqrt{a}\right]}{\sqrt{a}}\right),\;a=\frac{\lambda^{3}\left(t\right)}{\lambda^{3}\left(\tau \right)}-1. \end{array}$$

For a permanently crosslinked network, the reference state is always λ=1, and then Equation (9) for the tensile force acting on an average crosslink reduces to:

(10) $$\begin{array}{ccc} \langle f(t)\rangle & = & \frac{1}{2nr_{i0}}\frac{GJ_{m}}{J_{m}-J\left(t;0\right)}\left(\lambda \left(t\right)+\frac{\text{ArcSinh}\left[\sqrt{\lambda^{3}\left(t\right)-1}\right]}{\sqrt{\lambda^{4}\left(t\right)-\lambda \left(t\right)}}\right). \end{array}$$

**Figure 2:**
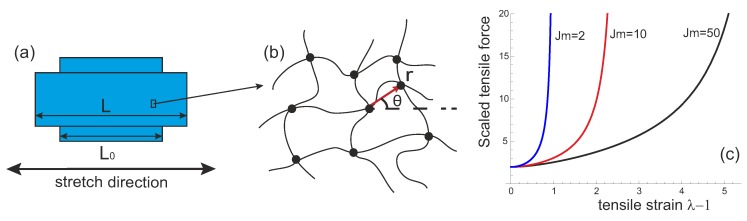
(**a**) a transient network undergoing a uniaxial stretch; (**b**) directions of the chains in the network with respect to the axis of uniaxial stretch; (**c**) scaled force $\left(2nr_{0}/G\right)\langle f\left(\lambda \right)\rangle$ from Equation (10) plotted for several values of stretchability limit $J_{m}$.

Taking the Gaussian chain limit gives $G=nk_{B}T$ and, using the average linear mesh size $r_{i0}=Nb^{2}/3$, we recover that the equilibrium network (at λ=1) has the average tension of its strands of $\langle f_{0}\rangle =k_{B}T/r_{i0}$. This is plotted in Figure 2c showing the equilibrium tension of equilibrium network, and its increase as the limit $J_{m}$ is approached.

### 2.4. Elastic Energy of Transient Network

The free energy of a transient network is more complex, as there are several contributions to the total energy of its different components. We again assume that a transient network was at the force-free state at time t=0, and is deformed at all time t>0. Then, the crosslinked chains at current time *t* can be divided into two classes: the chains surviving from the original time t=0, with a diminishing number $N_{\mathrm{sc}}\left(t\right)$, and the chains surviving from the time when they were newly crosslinked at a time $0<t^{\prime }<t$, with a dynamic number $N_{\mathrm{nc}}\left(t\right)$. For the originally crosslinked surviving chains, their contributions to the energy density of the system can be written proportionally as $N_{\mathrm{sc}}/N_{c}\left(0\right)\cdot F_{\mathrm{Gent}}\left(t;0\right)$. For the surviving chains that were crosslinked at an intermediate time $t^{\prime }$ during the deformation process, the energy contribution is more complex, because they have different reference states of deformation. We assume the elastic free energy density is additive (as was the underlying assumption in all classical rubber-elastic models) and proportional to the number of crosslinked chains. For example, if there is a population of $N_{\mathrm{nc}}\left(t_{0}\right)$ chains crosslinked at time $t_{0}$, then the proportional contribution of these chains to the energy density of the whole system should be $N_{\mathrm{nc}}\left(t_{0}\right)/N_{c}\left(0\right)\cdot F_{\mathrm{Gent}}\left(t;t_{0}\right)$, with their reference state at the time $t_{0}$. The energy density $F_{\mathrm{Gent}}\left(t;t_{0}\right)$ is a function of the deformation tensor $E(t;t_{0})$, with the reference state at the same time $t_{0}$: $E\left(t;t_{0}\right)=E\left(t;0\right)E^{-1}\left(t_{0};0\right)$.

Summing up the contributions from all chains re-crosslinked from t=0 to *t*, together with that from the $N_{\mathrm{sc}}\left(t\right)$ surviving chains crosslinked at the beginning, the total elastic energy of the transient network takes the following form:

(11) $$\begin{array}{ccc} F_{\mathrm{tr}.n.}\left(t\right) & = & e^{-\int_{0}^{t}\beta \left(t^{\prime };0\right)dt^{\prime }}F_{\mathrm{Gent}}\left(t;0\right)+\int_{0}^{t}\rho \frac{N_{b}\left(t^{\prime }\right)}{N_{c}\left(0\right)}e^{-\int_{t^{\prime }}^{t}\beta \left(t^{\prime \prime },t^{\prime }\right)dt^{\prime \prime }}F_{\mathrm{Gent}}\left(t;t^{\prime }\right)dt^{\prime }. \end{array}$$

The total stress of system capable of plastic flow has the elastic and the viscous parts. The origins of the viscous stress can be complex, including the nonaffine movements of the crosslinks, the dynamics of the dangling chains and the entanglements, and certainly the pair-correlation effects that determine viscosity of liquids according to Zwanzig and Kirkwood, see [34,35]. We are not going to be concerned with this part, and will simply write it as $\sigma^{\mathrm{vis}}=\eta \left(\dot{\gamma }\right)\cdot \dot{\gamma }$, where η is the appropriate friction tensor, expressed as a function of the strain rate tensor $\dot{\gamma }$. A large amount of literature exists on how the viscous stress depends on the strain rate, such as shear thickening and shear thinning [36,37] phenomena, which are usually explained by nonaffine movements in the network.

Here, we concentrate on how the elastic stress of the transient network changes dynamically when being deformed. For simplicity, the transient network is treated as incompressible. There are several ways to incorporate the condition of incompressibility, and we will introduce a Lagrangian multiplier to account for the volume constraint. Based on the Helmholtz free energy density obtained in Equation (11), the corresponding Gibbs free energy density of the system takes the form

(12) $$\begin{array}{ccc} g(t) & = & F_{\mathrm{tr}.n.}\left(t\right)-p\cdot \det E, \end{array}$$

where *p* is the Lagrangian multiplier in charge of the imposed incompressibility condition, with the same dimensions as pressure. The engineering elastic stress is evaluated with respect to the initial reference state at t=0,

(13) $$\begin{array}{c} \sigma_{ij}^{\mathrm{ela}}\left(t\right)=\frac{\delta g(t)}{\delta E_{ij}\left(t;0\right)} \end{array}$$

and the resulting constitutive relation takes the form

(14) $$\begin{array}{ccc} \sigma^{\mathrm{ela}} & = & GJ_{m}e^{-\int_{0}^{t}\beta \left(t^{\prime };0\right)dt^{\prime }}\frac{E(t;0)}{J_{m}-J\left(t;0\right)}+GJ_{m}\int_{0}^{t}\rho \frac{N_{b}\left(t^{\prime }\right)}{N_{0}}e^{-\int_{t^{\prime }}^{t}\beta \left(t^{\prime \prime },t^{\prime }\right)dt^{\prime \prime }}\frac{E\left(t;t^{\prime }\right){\left[E(t^{\prime };0)\right]}^{-T}}{J_{m}-J\left(t;t^{\prime }\right)}dt^{\prime }\\ & & -p{\left[E(t;0)\right]}^{-T}, \end{array}$$

where the first term represents the stress arising from the crosslinks surviving from the beginning, the second term reflects the stress originating from the surviving crosslinks formed during the deformation process, while the value of the Lagrangian multiplier *p* needs to be obtained from the boundary conditions of the material in specific deformation geometries.

## 3. Deformation of Uniaxial Extension

Now, we concentrate on a specific case, where the network is stretched uniaxially. It is a commonly applied method for studying dynamic mechanical response of viscoelastic materials. There are several characteristic phenomena found in this experimental geometry, including yield (stress overshoot) and a “neck” formation. Theoretically, the yield of a transient network is relatively well understood; it happens when most of the crosslinks break and reform without memorizing the deformation history. A theory of necking instability was recently proposed for a dual network gel [27,29,30], where the physical reason is thought to be the competition between the plasticity of the transient and the elasticity of the permanent network components. Necking phenomena appear in other systems, such as plastics and metals, but the defining factors there can be different and usually depend on specific interactions between molecules or atoms in different materials.

Let us consider an elastic network uniaxially stretched along *z* axis, with the stretching ratio λ(t) measured from its reference state at time t=0. The incompressible deformation tensor takes the form:

(15) $$\begin{array}{ccc} E(t;0) & = & \frac{1}{\sqrt{\lambda (t)}}\left(e_{x}e_{x}+e_{y}e_{y}\right)+\lambda \left(t\right)e_{z}e_{z}. \end{array}$$

By inserting this deformation tensor into Equation (14), the constitutive relations for the principal components of engineering stress along and perpendicular to *z* can be expressed as:

(16) $$\begin{array}{ccc} \sigma_{x,y} & = & \frac{GJ_{m}}{J_{m}-J\left(t;0\right)}\frac{1}{\sqrt{\lambda (t)}}e^{-\int_{0}^{t}\beta \left(t^{\prime };0\right)dt^{\prime }}+\int_{0}^{t}\rho \frac{GJ_{m}}{J_{m}-J\left(t;t^{\prime }\right)}\frac{\lambda (t^{\prime })}{\sqrt{\lambda (t)}}\frac{N_{b}\left(t^{\prime }\right)}{N_{0}}e^{-\int_{t^{\prime }}^{t}\beta \left(t^{\prime \prime },t^{\prime }\right)dt^{\prime \prime }}dt^{\prime }\\ & & \;-p\sqrt{\lambda (t)}, \end{array}$$

(17) $$\begin{array}{ccc} \sigma_{z} & = & \frac{GJ_{m}}{J_{m}-J\left(t;0\right)}\lambda \left(t\right)e^{-\int_{0}^{t}\beta \left(t^{\prime };0\right)dt^{\prime }}+\int_{0}^{t}\rho \frac{GJ_{m}}{J_{m}-J\left(t;t^{\prime }\right)}\frac{\lambda (t)}{\lambda {\left(t^{\prime }\right)}^{2}}\frac{N_{b}\left(t^{\prime }\right)}{N_{0}}e^{-\int_{t^{\prime }}^{t}\beta \left(t^{\prime \prime },t^{\prime }\right)dt^{\prime \prime }}dt^{\prime }\\ & & \;-p/\lambda (t). \end{array}$$

In the uniaxially stretched sample unconstrained from perpendicular directions, the stresses along *x* and *y* axes should be zero. This defines the value of Lagrange multiplier *p*, and then the tensile engineering stress along *z* axis is obtained by substituting p(λ) and grouping similar terms:

(18) $$\begin{array}{ccc} \sigma_{z} & = & \;\frac{GJ_{m}}{J_{m}-J\left(t;0\right)}\left(\lambda \left(t\right)-\frac{1}{\lambda {\left(t\right)}^{2}}\right)e^{-\int_{0}^{t}\beta \left(t^{\prime };0\right)dt^{\prime }}\\ & & +\int_{0}^{t}\rho \frac{GJ_{m}}{J_{m}-J\left(t;t^{\prime }\right)}\left(\frac{\lambda (t)}{\lambda {\left(t^{\prime }\right)}^{2}}-\frac{\lambda (t^{\prime })}{\lambda {\left(t\right)}^{2}}\right)\frac{N_{b}\left(t^{\prime }\right)}{N_{0}}e^{-\int_{t^{\prime }}^{t}\beta \left(t^{\prime \prime },t^{\prime }\right)dt^{\prime \prime }}dt^{\prime }. \end{array}$$

With this constitutive relation, we now can study how the dynamic mechanical response of a transient network, depends on an applied strain amplitude and rate, e.g., during a linear-ramp deformation with the stretching ratio $\lambda \left(t\right)=1+\dot{\gamma }t$. Note that this system has an internal characteristic time scale, equal to $1/\beta_{0}$, and both the rate of re-crosslinking and the rate of imposed strain need to be measured against that scale. Note that the true tensile stress along *z* (defined as the tensile force per unit area of current sample cross-section) is equal to $\lambda \sigma_{z}$ in an incompressible system. In the infinitesimal deformation limit of a fixed rubbery network, at ε=λ-1→0, this true stress can be approximated as $G(\lambda^{2}-1/\lambda )\approx 3G\varepsilon$, equal to the engineering stress $G(\lambda -1/\lambda^{2})\approx 3G\varepsilon$, defining the linear Young modulus Y=3G.

It is difficult to evaluate the multiple nested time-integrals in the Equation (18). For a straightforward numerical calculation, a small time interval Δt is chosen (using the bare breaking rate $\beta_{0}$ as the dimensional time scale). Then, the stress along the stretching direction at a time t=NΔt can be obtained by discretising the expression:

(19) $$\begin{array}{ccc} \sigma_{z} & = & \frac{GJ_{m}}{J_{m}-J\left(N\Delta t;0\right)}\left(\lambda_{N\Delta t}-\frac{1}{\lambda_{N\Delta t}^{2}}\right)e^{-\sum_{i=1}^{N}\beta \left(i\Delta t;0\right)\Delta t}\\ & & +\sum_{j=0}^{N-1}\frac{GJ_{m}}{J_{m}-J\left(N\Delta t;\left(j+1\right)\Delta t\right)}\left(\frac{\lambda_{N\Delta t}}{\lambda_{(j+1)\Delta t}^{2}}-\frac{\lambda_{(j+1)\Delta t}}{\lambda_{N\Delta t}^{2}}\right)\frac{N_{b}\left(j\Delta t\right)\rho }{N_{0}}\;e^{-\sum_{k=2+j}^{N}\beta \left[k\Delta t,\left(j+1\right)\Delta t\right]\Delta t}\Delta t. \end{array}$$

For example, after the first time step Δt, the stretch ratio along the stretch direction of the material becomes $\lambda_{\Delta t}=1+\dot{\gamma }\Delta t$. Correspondingly, the first strain invariant for the material with the reference state at time t=0 is $J\left(\Delta t;0\right)=\lambda_{\Delta t}^{2}+2/\lambda_{\Delta t}$. The ratio of the chains remaining crosslinked from the time t=0 to t=Δt is $e^{-\beta (\Delta t,0)\Delta t}$, which can be obtained from Equation (2). In this interval of time, there are $N_{b}\rho \Delta t/N_{0}$ newly crosslinked chains established, for which the reference state is set at the time Δt. Since, under our assumptions, the newly crosslinked chains do not contribute to stress, the stress $\sigma_{z}$ after the first time step is: $GJ_{m}\left(\lambda_{\Delta t}-1/\lambda_{\Delta t}^{2}\right)e^{-\beta (\Delta t;0)\Delta t}/\left(J_{m}-J\left(\Delta t;0\right)\right)$. After another time step, Δt the stretch ratio of the system becomes $\lambda_{2\Delta t}=1+2\dot{\gamma }\Delta t$, and the first strain invariant of the material with the reference state at t=0 is $J\left(2\Delta t;0\right)=\lambda_{2\Delta t}^{2}+2/\lambda_{2\Delta t}$. For the fraction of chains with the reference state at Δt, the first strain invariant becomes $J\left(2\Delta t;\Delta t\right)=\lambda_{2\Delta t}^{2}/\lambda_{\Delta t}^{2}+2\lambda_{\Delta t}/\lambda_{2\Delta t}$. Note that the chains crosslinked at t=Δt now start to contribute to the total stress, by the amount: $\rho GJ_{m}/\left(J_{m}-J\left(2\Delta t;\Delta t\right)\right)\left(\lambda_{2\Delta t}/\lambda_{\Delta t}^{2}-\lambda_{\Delta t}/\lambda_{2\Delta t}^{2}\right)N_{b}\left(\Delta t\right)\Delta te^{-\beta (2\Delta t,\Delta t)\Delta t}/N_{0}$. At the same time, the stress contribution of the crosslinked chains surviving from t=0 to t=2Δt decreases to: $GJ_{m}\left(\lambda_{2\Delta t}-1/\lambda_{2\Delta t}^{2}\right)e^{-\beta (\Delta t;0)\Delta t-\beta (2\Delta t;0)\Delta t}/\left(J_{m}-J\left(2\Delta t;0\right)\right)$. Adding these two contributions, we can obtain the stress $\sigma_{z}\left(2\Delta t\right)$ and move to the next time step in Equation (19).

**Figure 3:**
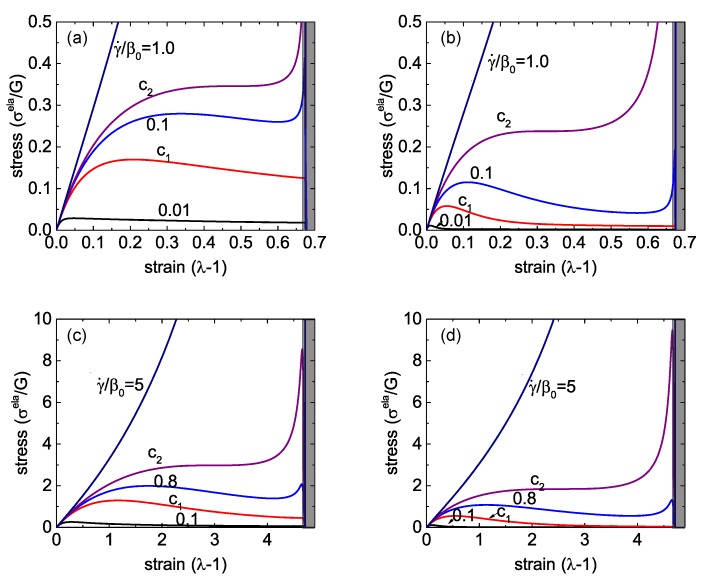
Stress-strain relations of a single transient network for different strain rates. (**a**) $J_{m}=1$ and $\rho /\beta_{0}=10$; (**b**) $J_{m}=1$ and $\rho /\beta_{0}=0.1$; (**c**) $J_{m}=30$ and $\rho /\beta_{0}=10$; (**d**) $J_{m}=30$ and $\rho /\beta_{0}=0.1$.

Stress-strain relationship in Equation (18) for different strain rates are shown in Figure 3, for several distinct sets of parameters. Firstly, we consider the case where the re-crosslink rate is much higher than the spontaneous rate of breaking, e.g., $\rho /\beta_{0}=10$, which means that the bond energy is greater than the barrier for attachment ($W_{b}>W_{c}$) and, in equilibrium, most of the network crosslinks are engaged. The stress-strain curves for this case are shown in Figure 3a for $J_{m}=1$, that is, when the limit of chain extension is quite low, and in Figure 3c for $J_{m}=30$, that is, when the elastomer network has a very large limit of extensibility (recall the definition of $J_{m}=\sum_{i}\lambda_{m,i}^{2}-3$, which is a fixed value defined with respect to the initial reference state of the network at t=0). The other pair of plots in Figure 3 are for the case $\rho /\beta_{0}=0.1$, that is, when the crosslinks are quite easy to break and take a long time to re-connect.

Qualitatively, the constitutive relationship has the same characteristic features in all these different cases. At infinitesimally low strains, there is a linear response $\sigma^{\mathrm{ela}}=3G\varepsilon$, where the rubber shear modulus *G* is the underlying parameter of the Gent model; it is used to scale the stress axis in all plots. When the strain rate $\dot{\gamma }$ of the imposed linear ramp is quite low (compared to the spontaneous rate of crosslink breaking $\beta_{0}$), we see a classic transition to the plastic-flow plateau with $\sigma^{\mathrm{ela}}$ decreasing and the viscous stress $\eta \dot{\gamma }$ becoming the only relevant contribution.

At a very high strain rate, the network remains strictly elastic with a monotonically growing $\sigma^{\mathrm{ela}}\left(\lambda \right)$. Below a critical value $\dot{\gamma }$, which we denote $c_{2}$ in all plots in Figure 3, the stress-strain curve develops an “overshoot”—a yield stress point past which the slope of the curve becomes negative. This is a distinctly dynamic feature: a signature of mechanical instability in the material. For a wide range of strain rates, this instability manifests itself as breaking the sample into two coexisting domains: one with a strain value in the elastic regime, before the yield point—the other in the strongly plastic regime, at large effective extension where the stress-strain makes another sharp upturn in $\sigma^{\mathrm{ela}}\left(\lambda \right)$ on approaching the Gent limit $J_{m}$ (in the shaded region in each plot) where the local force on each crosslink diverging near $J_{m}$ results in all crosslinks breaking.

**Figure 4:**
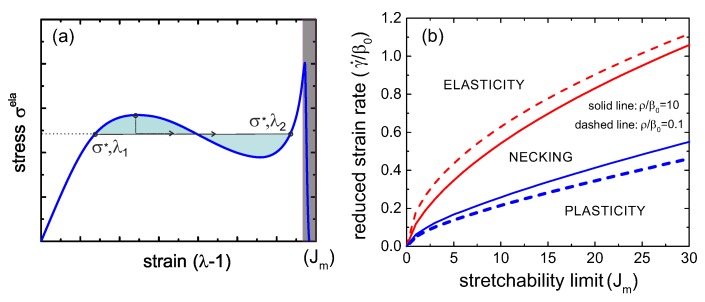
(**a**) The scheme of the necking instability tie line connecting the states with the same linear force: $\left(\sigma^{*},\;\lambda_{1}\right)$ and $\left(\sigma^{*},\;\lambda_{2}\right)$, with the equal-area condition defining the level $\sigma^{*}$; (**b**) the phase diagram (in variables $J_{m},\dot{\gamma }$) of the mechanical response in a transient network undergoing uniaxial stretching with a linear ramp, comparing the cases $\rho /\beta_{0}=10$ (**solid lines**)and 0.1 (**dashed lines**).

The balance of forces is the criterion to determine the tie line of this necking instability. Since the main equation in the uniaxial case, Equation (18), represents the engineering tensile stress, and the force is a product of this stress and the initial (un-distorted) cross-section area, the equality of forces means the equality of engineering stress (the balance, and the tie line, will look a bit different in the true stress representation). Hence, the tie line is horizontal, connecting the points $\left(\sigma^{*},\;\lambda_{1}\right)$ and $\left(\sigma^{*},\;\lambda_{2}\right)$, see Figure 4a for illustration. If we employ the quasi-equilibrium condition for coexistence of the two regions, *i.e.*, the equality of the Gibbs free energy density $g\left(\lambda_{1}\right)=g\left(\lambda_{2}\right)$, then the connection $g_{2}=g_{1}+\int_{1}^{2}\sigma \;d\lambda$ shows that the condition of equal areas determines the position of the tie line $\sigma^{*}$.

However, since we are considering a dynamically increasing strain, the actual transition will be determined by the energy barriers. The lower bound (for smallest energy barrier, which is essentially determined by the energy of interface between $\lambda_{1}$ and $\lambda_{2}$ regions) is the tie line itself. That is, on increasing the strain (at a fixed rate $\dot{\gamma }$) the sample reaches the point $\left(\sigma^{*},\;\lambda_{1}\right)$, and the infinitesimal region of $\lambda_{2}$ emerges, coexisting with $\lambda_{1}$. The fraction of $\lambda_{2}$ will increase according to the linear superposition $x\;\lambda_{2}+\left(1-x\right)\lambda_{1}=\lambda$ until the point $\left(\sigma^{*},\;\lambda_{2}\right)$ is reached along the tie line, after which the sample will return to the homogeneous highly stretched state. The upper bound (for significant energy barriers) will be when the samples ‘overshoots’ the point $\left(\sigma^{*},\;\lambda_{1}\right)$ and reaches the upper limit of stability, where the negative slope of σ(λ) first appears. Then, the sample will have a discontinuous jump to a finite fraction of the highly-stretched $\lambda_{2}$ region along the tie line. The highest shear rate at which this instability is possible is labelled $c_{2}$ in Figure 3. Meanwhile, the lower limit of stability of the coexistence (necking) regime is the curve marked $c_{1}$ in Figure 3, where the upturn of the $\sigma^{\mathrm{ela}}\left(\lambda \right)$ relationship starts at large deformation. The reason for this upturn is that there are more crosslinked chains surviving from the beginning for larger strain rates, and they contribute to the rapid increase of the total stress when these chains experience the hyper-nonlinear region of the Gent model on J(λ) approaching its limit $J_{m}$. At strain rates below $\dot{\gamma }\left(c_{1}\right)$, the stress monotonically decreases at high strains.

Stress turnovers can disappear at too-low and too-high strain rates. Figure 4b gives a “phase diagram” of the process as a map in the space of $\left(J_{m},\dot{\gamma }\right)$ parameters, marking the regions of pure elasticity, of pure plastic flow above the yield stress, and of the two-phase necking regime. Curiously, we see again (as in Figure 3) that, in spite of a very different appearance of networks with high and low rates of re-crosslinking, the actual difference in their behavior is quite minor. The critical values of the strain rate, $\dot{\gamma }\left(c_{1}\right)$ and $\dot{\gamma }\left(c_{2}\right)$, both increase with the stretchability limit of the network $J_{m}$, eventually becoming linear at large $J_{m}$. This means that a transient network with no finite-extensibility limit ($J_{m}\to \infty$) is always in the plastic regime above the yield stress.

## 4. Dual Transient Network

Dual networks become more and more relevant in various areas of science and engineering. In biological networks, this is the key mechanism to achieve the required mechanical characteristics: in the cytoskeleton, the actin filament network is embedded in the weak intermediate filament gel, and in the extracellular matrix and connective tissue, one always finds a stiffer (usually collagen) network embedded in a much weaker gel of elastin, fibronectin, or another flexible protein [39]. In synthetic elastomers, the dual interpenetrating network concept is very widely spread due to the remarkable mechanical properties and the resilience of the material [40].

We now consider a case of dual transient network, as an extension of the earlier general analysis. Two different kinds of elastomeric networks penetrating each other share the common affine deformation and add the forces generated in response; we shall denote the variables of each network by their superscript: 1*st* or 2*nd*. The Helmholtz free energy density of the system is the sum of the contributions from each network, and the corresponding Gibbs free energy density follows:

(20) $$\begin{array}{c} F_{\mathrm{tr}.n.}\left(t\right)=F_{\mathrm{tr}.n.}^{1st}\left(t\right)+F_{\mathrm{tr}.n.}^{2nd}\left(t\right);\;\;\;\mathrm{with}\;\;\;g\left(t\right)=F_{\mathrm{tr}.n.}\left(t\right)-p\cdot \det E. \end{array}$$

Then, the engineering elastic stress can be obtained from Equation (13), which can also be expressed as a sum of the two contributions, $\sigma^{1st}+\sigma^{2nd}$ (since the Lagrange multiplier could be split between the two parts, as in the partial pressure).

Although it is not necessary, let us consider the basic rubber elasticity of the two networks to be the same (the same rubber modulus *G*), since our main interest is in understanding the dynamics of the response. Similarly, for understanding qualitative features of network dynamics, let us assume that both components of the dual system have the same limit of the stretchability $J_{m}$. We consider the difference between the two networks to be in the microscopic dynamics of their crosslinks: the crosslink breakage rates. One can easily adapt the general theoretical model for any specific system by using Equation (20), and the particular parameters of each individual network as given explicitly in the previous section. Note that since we are considering the imposed strain (rather than stress), the local force applied to each individual crosslink remains the same, as given by Equation (9), irrespective of whether the other component network is breaking or not.

The two different breaking rates of the component networks are $\beta_{0}^{1st}$ and $\beta_{0}^{2nd}$, each modified by the additional exponential factor with the local pulling force. If one breakage rate is much smaller than the other, that component can be regarded as permanent elastic network.

**Figure 5:**
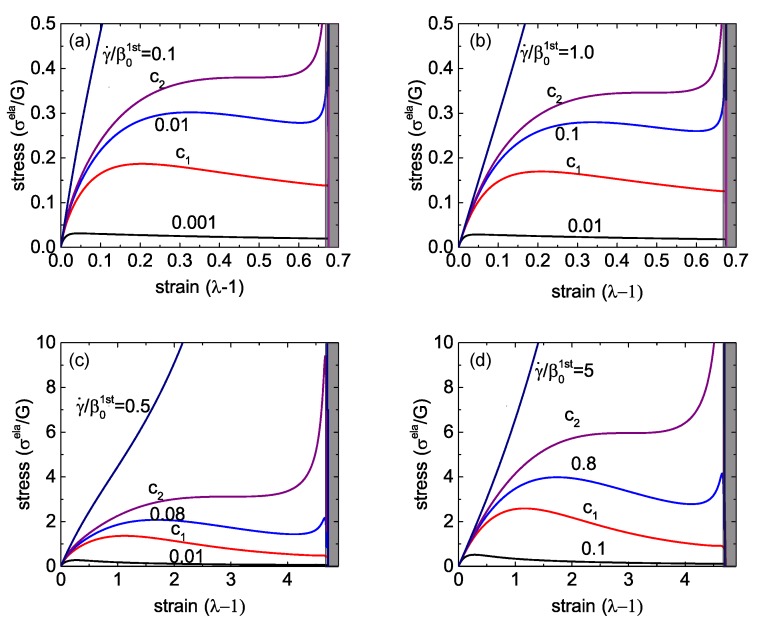
Stress-strain relations of a single transient network for different strain rates. (**a**) $J_{m}=1$ and $\beta_{0}^{2nd}/\beta_{0}^{1st}=0.1$; (**b**) $J_{m}=1$ and $\beta_{0}^{2nd}/\beta_{0}^{1st}=1$; (**c**) $J_{m}=30$ and $\beta_{0}^{2nd}/\beta_{0}^{1st}=0.1$; (**d**) $J_{m}=30$ and $\beta_{0}^{2nd}/\beta_{0}^{1st}=1$.

**Figure 6:**
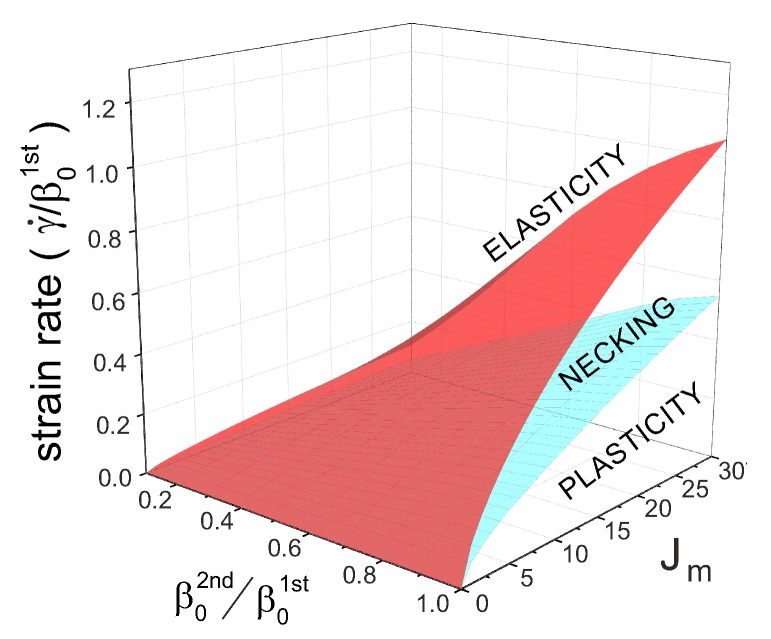
Phase diagram in a dual network undergoing a uniaxial stretch, where the characteristic time scale is chosen as: (a) $1/\beta_{0}^{1st}$, and (b) $1/\beta_{0}^{2nd}$. The cross-section plane of $\beta_{0}^{2nd}/\beta_{0}^{1st}=1$ is the plot in Figure 4; the cross-section plane of $\beta_{0}^{2nd}=0$ corresponds to the permanent second network , and both phase boundaries collapse to zero leaving only the elastic response available.

Similar to the case of a single transient network, here we will discuss the responses of a dual network to a uniaxial stretch by a fixed factor λ in the form of the linear ramp deformation $\lambda =1+\dot{\gamma }\;t$. As in the plots in Figure 3 and Figure 4b, we scale the time in the units of $1/\beta_{0}^{1st}$. Two important physical variables determine the stress-strain relationship of a dual network: the reduced strain rate, $\dot{\gamma }/\beta_{0}^{1st}$, and the reduced breakage rate of the crosslinks of the second network at its force free state, $\beta_{0}^{2nd}/\beta_{0}^{1st}$. Figure 5 shows the three deformation regimes of plasticity, elasticity and necking, similar to the single transient network discussion earlier. If the stretchability limit of the material is small, say $J_{m}=1$ in Figure 5a,b, the critical values of the reduced strain rate, $c_{1}$ and $c_{2}$, scale in proportion to the ratio $\beta_{0}^{2nd}/\beta_{0}^{1st}$. Only two cases of this ratio =0.1 and =1 are shown, but the relation holds in general, as the 3D phase diagram in Figure 6 demonstrates. This indicates that the macroscopic response, or specifically—the boundaries of three characteristic regimes of a dual network—is mainly determined by the more permanent (or less breakable) component. Note that, since we add the two stress components, there is an inherent symmetry, *i.e.*, if the ratio $\beta_{0}^{2nd}/\beta_{0}^{1st}$ becomes greater than 1, then the 1st network becomes “more permanent” and then it determines the phase boundaries.

The plots in Figure 5c,d compare with the case of a much less restricted network: the limit of stretchability $J_{m}=30$. The critical values $c_{1}$ and $c_{2}$ of the reduced strain rate $\dot{\gamma }/\beta_{0}^{1st}$ increase with the stretchability limit of the material, similar to what we observed in a single transient network, while the proportionality to the rates ration remains valid.

Figure 6 shows a ‘phase diagram’ of the regions of elasticity, necking and plastic flow, exploring the effects of stretchability limit $J_{m}$, the bare rates ratio $\beta_{0}^{2nd}/\beta_{0}^{1st}$, and the rate of strain. Note that the plane of $\beta_{0}^{2nd}=0$ is the case of the second network component remaining permanent—and we see in Figure 6 that the dual network will remain elastic at all rates of strain. This is in contrast to the earlier work [29], which is because the authors there assumed that the proportion of the second permanent network is ϕ=0.1, so it has small effect and still allows for necking—whereas we have considered the symmetric case of two networks in equal proportion, and find that the elasticity prevails when one of the networks is unbreakable. Similarly, the plane $\beta_{0}^{2nd}=\beta_{0}^{1st}$ is the case of single transient network we discussed earlier, and the cross-section in this plane is our old Figure 4b. We have already discussed in the context of a single network, that at large $J_{m}$ (that is, when Gent model reduces to the classical rubber elasticity) both phase boundaries depend linearly on $J_{m}$, and this remains the case in the dual network.

## 5. Conclusions

In this work, we developed a consistent theory for handling a transient network undergoing a finite/large deformation, where the network strands could be stretched to their limit, and where the microscopic dynamics of crosslinks under tensions and the macroscopic deformation of the network are bridged in a unified framework. By applying this theory, three deformation regimes are identified: plastic flow, necking instability and elastic response, with sharp transition boundaries between them. For a dual transient network system, the response of the system to the deformation mainly depends on the component with a smaller breakage rate.

Note that there exists a closely related phenomenon: shear banding, which is found when a system with complex nonlinear rheological signature is deformed [38]. Shear banding instability occurs due to the nonlinearity arising from the coupling between the flow and the changes in mesoscopic structure, resulting in two states coexisting with different shear rates at a common shear stress. In our system, the necking instability (the coexistence of two states with different local strain at a common tensile stress) is induced by a uniaxial stretch is imposed on a transient elastic network, due to a competition between the hyper-elasticity of the chains and the plasticity introduced by the breakable crosslinks.

Non-affine movements of crosslinks in a random network might become relevant when deforming a transient network with low-density of crosslinks or many geometric entanglements that can be released; these will lower the effective stress in the network under a fixed extension. We also did not consider non-homogeneity in the structure of the network, e.g., in the crosslink density, while it could be important to produce high-strength materials. On the positive side, this theoretical model is very portable and can be adapted for many systems, such as cytoskeleton networks and extracellular matrix, where the proposed mechanism for three deformation regimes should be qualitatively valid.
